# Antimicrobial Resistance Pattern, Pathogenicity and Molecular Properties of Hypervirulent *Klebsiella pneumonia* (hvKp) among Hospital-Acquired Infections in the Intensive Care Unit (ICU)

**DOI:** 10.3390/microorganisms11030661

**Published:** 2023-03-06

**Authors:** Mohanned Talal Alharbi, Mohammed S. Almuhayawi, Mohammed K. Nagshabandi, Muyassar K. Tarabulsi, Mohammed H. Alruhaili, Hattan S. Gattan, Soad K. Al Jaouni, Samy Selim, Awadh Alanazi, Yasir Alruwaili, Shaimaa Mohamed Zaied, Osama Ahmed Faried

**Affiliations:** 1 Department of Medical Microbiology and Parasitology, Faculty of Medicine, University of Jeddah, Jeddah 23218, Saudi Arabia; 2 Department of Medical Microbiology and Parasitology, Faculty of Medicine, King AbdulAziz University, Jeddah 21589, Saudi Arabia; 3 Special Infectious Agents Unit, King Fahad Medical Research Center, King AbdulAziz University, Jeddah 21589, Saudi Arabia; 4 Department of Medical Laboratory Technology, Faculty of Applied Medical Sciences, King Abdulaziz University, Jeddah 21589, Saudi Arabia; 5 Department of Hematology/Oncology, Yousef Abdulatif Jameel Scientific Chair of Prophetic Medicine Application, Faculty of Medicine, King Abdulaziz University, Jeddah 21589, Saudi Arabia; 6 Department of Clinical Laboratory Sciences, College of Applied Medical Sciences, Jouf University, Sakaka 72341, Saudi Arabia; 7 Clinical and Chemical Pathology Department, Faculty of Medicine, Beni-Suef University, Beni-Suef 62513, Egypt; 8 Medical Microbiology and Immunology Department, Faculty of Medicine, Beni-Suef University, Beni-Suef 62513, Egypt

**Keywords:** hospital-acquired, hypervirulent, *Klebsiella pneumonia*, hvKp, intensive care unit, emergence, antimicrobial resistance, Saudi Arabia

## Abstract

Hypervirulent *Klebsiella pneumoniae* (hvKp) is a new emerging variant of *K. pneumoniae* that is increasingly reported worldwide. The variant hvKp is known to cause severe invasive community-acquired infections such as metastatic meningitis, pyogenic liver abscesses (PLA) and endophthalmitis, but its role in hospital-acquired infections (HAIs) is little known. The aim of this study was to evaluate the prevalence of hvKp among hospital-acquired (HA) *K. pneumoniae* infections in the intensive care unit (ICU) and to compare between hvKp and classical *K. pneumoniae* (cKP) regarding antimicrobial resistance pattern, virulence and molecular characteristics. The study was cross-sectional and included 120 ICU patients suffering from HA *K. pneumoniae* infections between January and September 2022. *K. pneumoniae* isolates were subjected to antimicrobial susceptibility testing and detection of extended-spectrum-β-lactamase (ESBL) production by the Phoenix 100 automated microbiology system, string test, biofilm formation, serum resistance assay, and detection of virulence-associated genes (*rmpA*, *rmpA2*, *magA*, *iucA)* and capsular serotype-specific genes (*K1*, *K2*, *K5*, *K20*, *K57)* by polymerase chain reaction (PCR). Of 120 *K. pneumoniae* isolates, 19 (15.8%) were hvKp. The hypermucoviscous phenotype was more significantly detected in the hvKp group than in the cKP group (100% vs. 7.9%, *p* ≤ 0.001). The rate of resistance to different antimicrobial agents was significantly higher in the cKP group than that in the hvKp group. Fifty-three strains were identified as ESBL-producing strains, which was more frequent in the cKP group than in the hvKp group (48/101 [47.5%] vs. 5/19 [26.3%], respectively, *p* ≤ 0.001). The hvKP isolates were highly associated with moderate and strong biofilm formation than cKP isolates (*p* = 0.018 and *p* = 0.043 respectively). Moreover, the hvKP isolates were highly associated with intermediate sensitivity and re sistance to serum in the serum resistance assay (*p* = 0.043 and *p* = 0.016 respectively). K1, K2, rmpA, rmpA2, magA and iucA genes were significantly associated with hvKp (*p* ≤ 0.001, 0.004, <0.001, <0.001, 0.037 and <0.001, respectively). However, *K5*, *K20* and *K57* were not associated with hvKp. The hvKp strains have emerged as a new threat to ICU patients because of their ability to cause more severe and life-threatening infections than cKP. The string test alone as a laboratory test for screening of hvKp has become insufficient. Recently, hvKp was defined as hypermucoviscous- and aerobactin-positive. It is important to improve the awareness towards the diagnosis and management of hvKp infections.

## 1. Introduction

*Klebsiella pneumoniae* (Kp) is a clinically important member of the *Enterobacteriaceae* family that can cause a wide range of infections such as pneumonia, urinary tract, bacteremia and wound infections. *K. pneumonae* usually cause hospital-acquired infections (HAIs) and occur primarily in patients with impaired immunity. *Klebsiella pneumoniae* has two different pathotypes: classical (cKp), which is the most common subtype of the *K.* pneumoniae strains that has received increased notoriety due to its ability to develop multidrug resistance and a new emerging variant termed hypervirulent (hvKp) [1,2,3]. The variant hvKp was first recognized in Taiwan in 1986 [4]. The hvKp strains are able to cause metastatic and life-threatening infections in immunocompetent and young healthy individuals. The variant hvKp is a leading cause of pyogenic liver abscesses (PLA) and it has also been implicated in metastatic meningitis and endophthalmitis. The variant hvKp has a characteristic hypermucoviscous colonies when grown on an agar plate, which can be detected by string test [5]. However, there are some controversies about the definition of hvKp. Many factors should be considered such as host, pathogen and host–pathogen interactions when defining hvKp. Nevertheless, most published papers have concentrated only on the bacteria. A previous study revealed that nutritional status, major histocompatibility complex (MHC) variants, composition of gut microbiota and eating habits are important host factors to investigate in order to improve our understanding of the hypervirulence phenomenon [6]. In addition, some controversies have arisen regarding the relationship between hypermucoviscous phenotype and virulence. Using in vivo and in vitro experiments, several studies revealed that few hypermucoviscous *K. pneumonia* (hmvKp) isolates are associated with high virulence [7,8]. In animal models, hmvKp did not show more serious infections or a higher mortality rate than non-hmvKp. Therefore, hvKp cannot be defined by string test alone [6,9].

Currently, aerobactin has been considered to be a crucial virulence factor for hvKp, which is often associated with the hypermucoviscous phenotype. Based on this finding, research conducted in China that included multiple centres first described the clinical and molecular characteristics of hvKp (defined as aerobactin-positive) isolates. The results revealed that invasive infections, especially PLA, hypermucoviscous phenotype and most of the virulence factors such as *rmp*A, *rmp*A2 (both regulators of the mucoid phenotype), and *mag*A (mucoviscosity-associated gene) and capsular serotype-specific genes (*K1*, *K2*), are strongly associated with aerobactin-positive Kp [9,10,11]. Furthermore, some studies have revealed that iron acquisition genes and the genes that encode the hypermucoviscous phenotype are situated on the same virulence plasmid, which is present in most hvKp isolates but rarely present in cKp strains [12,13,14]. Thus, it may be more appropriate to combine aerobactin positivity and hypermucoviscosity when defining hvKp.

This study was conducted to evaluate the prevalence of hvKp among hospital-acquired (HA) *K. pneumoniae* infections in the intensive care unit (ICU) and to compare hvKp and classical *K. pneumoniae* (cKp) regarding antimicrobial resistance pattern, virulence and molecular characteristics.

## 2. Methods

### 2.1. Patients

The study was cross-sectional and included 120 ICU patients suffering from HA *K. pneumoniae* infections in Prince Mutaeb Bin Abdulaziz Hospital, Aljouf, Kingdom of Saudi Arabia between January and September 2022. Duplicate isolates from the same patient were excluded. Infections were considered as HA when a new infection developed 48 h after patient admission. 

### 2.2. Clinical K. pneumoniae Isolates 

The strains were isolated from the following clinical specimens: respiratory secretions, urine, blood and wound swabs. The strains were identified by the Phoenix 100 automated microbiology system (BD, Franklin Lakes, NJ, USA). The hvKp strains were defined as hypermucoviscous- and aerobactin-positive, which was confirmed by string test and PCR, respectively. The strains were stored on glycerol at −80 °C until PCR was performed. 

### 2.3. Antibiotic Susceptibility Testing and Detection of ESBL Production

The susceptibility of the isolates to clinically relevant antibiotics (amikacin, gentamycin, imipenem, meropenem, cefoxitin, ceftazidime, ceftriaxone, cefepime, aztreonam, ampicillin, amoxicillin- clavulanate, piperacillin-tazobactam, trimethoprime-sulphamethoxazole, ciprofloxacin, levofloxacin) and screening for extended-spectrum β-lactamase (ESBL) production were determined using the Phoenix 100 automated microbiology system. 

### 2.4. String Test

Bacterial colonies grown overnight on a blood agar plate at 37 °C were stretched by a standard bacteriological loop. If a mucoviscous string > 5 mm in length was formed, the string test was considered positive and the isolate was identified as hypermucoviscous [15].

### 2.5. Biofilm Formation

Biofilm formation was examined by using the semi-quantitative assay in 96-well flat bottom plates as previously described [16]. 

### 2.6. Serum Resistance Assay

The serum resistance assay was performed as previously described [15]. Then, viable counts (VCs) of bacteria were determined for a period of 3 h. Responses were classified into six grades as follows: highly sensitive (grade 1 and 2), intermediately sensitive (grade 3 and 4), or resistant (grade 5 and 6) [17].

### 2.7. Detection of Capsular Serotype-Specific Genes and Virulence-Associated Genes 

Detection of capsular serotype-specific genes (*K1*, *K2*, *K5*, *K20* and *K57)* and virulence-associated genes (*rmpA*, *rmpA2*, *magA* and *iucA*) were performed by polymerase chain reaction (PCR). The presence of the *iucA* gene was used to identify hvKp.

All isolated *K. pneumoniae* strains were subjected to DNA extraction by the boiling method as previously described [18]. The PCR used for amplification of capsular serotype-specific genes (*K1*, *K2*, *K5*, *K20* and *K57*) was conducted as previously described [19]. PCR for detection of virulence-associated genes (*rmpA*, *rmpA2* and *magA*) was applied as previously described [20,21]. PCR conditions for amplification of *iucA* (aerobactin) were as follows: initial denaturation at 95 °C for 15 min, followed by denaturation at 95 °C for 15 s, annealing at 49 °C for 15 min and extension at 72 °C for 1 min for 30 cycles followed by a final extension at 72 °C for 10 min. Primers used in this study are listed in Table 1. Then, the PCR products were subjected to electrophoresis at 100 V for 2 h in a 2% agarose gel containing ethidium bromide (0.5 µg/mL). DNA bands were visualized by UV illumination at 302 nm on a UV transilluminator.

### 2.8. Statistical Analysis

Statistical Package for Social Sciences (SPSS) version 20 was used for statistical analysis of the data. Qualitative data were described as numbers and percentages. Comparisons between groups were performed by chi-square test or Fisher’s exact test. Test results with *p* < 0.05 were considered significant.

## 3. Results

### 3.1. Patient Characteristics

This study included 120 ICU patients who were suffering from HA *K. pneumoniae* infections in Prince Mutaeb Bin Abdulaziz Hospital, Aljouf, Kingdom of Saudi Arabia. Sixty-six (55%) patients were males and 54 (45%) were females; the mean age was 51.78 ± 1.56 (mean ± SE) years. HvKP were defined as hypermucoviscous- and aerobactin-positive isolates were confirmed by string test and PCR, respectively. Nineteen out of one hundred twenty (15.8%) isolates were hvKp. The strains were isolated from the clinical specimens as follows: 48 (40%) from respiratory secretions (hvKp 7.5%, cKp 32.5%), 38 (31.66%) from urine (hvKp 6.66%, cKp 25%), 18 (15%) from blood (hvKp 0%, cKp 15%) and 16 (13.3%) from wound (hvKp 1.66%, cKp 11.66%). A significantly higher number of patients with cKp in respiratory secretions was detected (*p* = 0.0241). Otherwise, no significant differences were detected in between the isolation of hvKp and cKp isolates in any of the specimen types. The mean age of hvKp-infected patients was significantly younger than that of cKp-infected patients (31.26 ± 0.47 years vs. 55.63 ± 1.43 years, respectively, *p* ≤ 0.001). Microbiological and genetic characteristics of hvKp isolates are shown in Table 2.

### 3.2. Comparison between hvKp and cKp Isolates Regarding Antimicrobial Susceptibility Testing and ESBL Production

The rate of resistance to antimicrobial agents was significantly higher in cKp than in hvKp strains, except ampicillin (all hvKp strains were resistant to ampicillin). Fifty-five strains were identified as ESBL-producing strains, which was more significantly detected in the cKp strains than in the hvKp strains (51/101 [50.5%] vs. 4/19 [20.05%], respectively, *p* = 0.018). The results of antibiotic susceptibility testing and ESBL production for hvKp and cKp are shown in Table 3.

### 3.3. Comparison between hvKP and cKP Isolates Regarding Biofilm Formation and Serum

The hypermucoviscous phenotype (based on the string test) was detected in 27 (22.5%) of all *K. pneumoniae* strains and this phenotype was more frequent in hvKp isolates than in cKp isolates (100% vs. 7.9%, *p* ≤ 0.001).

### 3.4. Comparison between non ESBL Producing hvKp and non ESBL Producing cKp Isolates Regarding Biofilm Formation and Serum Resistance

The hvKp isolates were highly associated with moderate and strong biofilm formation than the cKp isolates (*p* = 0.018 and *p* = 0.043, respectively). Moreover, the hvKp isolates were highly associated with intermediate sensitivity and resistance to serum in the serum resistance assay (*p* = 0.043 and *p* = 0.016, respectively). Results are shown in Table 4.

### 3.5. Comparison between non ESBL Producing hvKP and ESBL Producing cKP Isolates Regarding Biofilm Formation

The hvKp isolates were highly associated with moderate biofilm formation and intermediate sensitivity to serum in the serum resistance assay than the ESBL-producing cKp isolates (*p* = 0.044 and *p* = 0.031, respectively). There were no significant differences with strong biofilm formation and resistance to serum in serum resistance assay between hvKp and ESBL-producing cKp isolates (*p* = 0.621 and *p* = 0.542, respectively). Results are shown in Table 5.

### 3.6. Comparison between ESBL-Producing hvKp and ESBL-Producing cKp Isolates Regarding Biofilm Formation and Serum Resistance

There was no significant difference between ESBL-producing hvKp and ESBL-producing cKp isolates regarding biofilm formation or serum resistance assay (all *p* > 0.05). Results are shown in Table 6.

### 3.7. Comparison between hvKp and cKp Isolates Regarding Genetic Characteristics

All isolated strains were tested for virulence-associated genes (*rmpA, rmpA2, magA* and aerobactin) and Capsular serotype-specific genes (*K1*, *K2*, *K5*, *K20* and *K57*) by PCR. In this study, *K1*, *K2*, *rmpA*, *rmpA2*, *magA* and aerobactin were significantly associated with hvKp strains (*p* ≤ 0.001, 0.004, <0.001, <0.001, 0.037 and <0.001, respectively). Nevertheless, *K5*, *K20* and *K57* were not significantly associated with hvKp strains.

### 3.8. Comparison between hvKp and cKp Isolates Regarding Risk Factors and Associated Invasive Infections

Diabetes mellitus was a statistically significant risk factor associated with hvKp isolates (78.9% in hvKp vs. 35.6% in cKp, *p* = 0.003). On the other hand, there were no significant differences between hvKp and cKp strains regarding other underlying conditions of patients. Invasive infections were more significantly detected in patients with hvKp infections than those with cKp (42.1% in hvKp vs. 4.9% in cKp, *p* ≤ 0.001). Differences between hvKp and cKp groups are shown in Table 7.

## 4. Discussion

The variant hvKp is an increasingly reported pathotype of *K. pneumoniae* characterized clinically by its capability to cause metastatic and life-threatening infections in immunocompetent and young healthy individuals. In our study, the hypermucoviscous phenotype (based on the string test) was identified in 22.5% of all *K. pneumoniae* strains and this phenotype was significantly higher in hvKp strains. Another study performed in Egypt reported that the hypermucoviscous phenotype was detected in about 40% of *K. pneumoniae* isolates [22]. This discrepancy may be due to the difference in sample size.

In this study, hvKp (identified as hypermucoviscous- and aerobactin-positive) accounted for 15.8% of all HA *K. pneumoniae* infections in ICUs. This is in line with previous studies that showed that the prevalence of hvKp ranged from 7.8% to 25.4 [23,24].

Our results showed that the mean age of hvKp-infected patients was significantly younger than that of cKp-infected patients and invasive infections were significantly higher in patients with hvKp infections. These results are consistent with previous reports that showed that hvKp frequently causes invasive infections in young people without underlying disease [25,26,27,28].

Regarding antimicrobial resistance and ESBL production, our results showed that the resistance rate to common antibiotics in hvKp strains was significantly lower than in cKp strains and that ESBL production was more frequent in the cKp strains than in the hvKp strains. This was in accordance with another study conducted in China that reported the same findings [29]. In another study, the population genomics data suggested that acquisition of antimicrobial resistance plasmids by hvKp was more difficult than in cKp. The authors postulated that this may be due to the hyper-expression of the capsule, which may provide a physical barrier against the acquisition of antimicrobial resistance plasmids [30]. However, another study conducted in Egypt reported that there was no significant difference between hvKp and cKp strains regarding the antimicrobial resistance pattern [31]. The reason for this discrepancy may be due to the difference in sample size.

Regarding biofilm formation and serum resistance assay, our study showed that the hvKp isolates were highly associated with moderate and strong biofilm formation than the cKp isolates. Moreover, the hvKp isolates were highly associated with intermediate sensitivity and resistance to the serum in serum resistance assay than the cKp isolates. This is in agreement with a previous study that reported the same findings [32]. In contrast, another study conducted in Egypt reported that there was no significant difference between hvKp and cKp regarding biofilm formation and serum resistance assay [31]. This discrepancy may be attributed to the difference in sample size.

Regarding the virulence factors, the capsule is considered the major virulence factor in K. *pneumoniae*; there are several types of K-antigens [33,34,35]. *K1* and *K2* are the most important serotypes as they frequently result in severe infections [36,37]. In this study, *K1* and *K2* were significantly higher in hvKp than in cKp strains. The genes responsible for the hypermucoviscous phenotype (*rmpA, mpA2* and *MagA*) are considered another virulence determinants in addition to K1/K2 [14,15,38,39]. Our results showed that *rmpA*, *rmpA2* and *magA* were significantly associated with hvKp strains. These results are consistent with a previous study, which reported that there was a significant association between these genes and hvKp but not with cKp [29]. Aerobactin is considered a crucial virulence determinant of hvKp. In agreement with another study, our results showed that aerobactin was significantly associated with hvKp [40]. Therefore, these results showed that most of the virulence factors are more strongly associated with hvKp than with cKp.

Aligning with the results of a previous study, our results revealed that diabetes mellitus was a statistically significant risk factor associated with hvKp infections and that there were no significant differences between hvKp and cKp strains regarding other underlying conditions of patients [31].

## 5. Conclusions

The hvKp strains have emerged as a new threat to ICU patients because of their ability to cause more severe and life-threatening infections than cKp. The string test alone as a laboratory test for screening of hvKp has become insufficient. Recently, hvKp has been defined as hypermucoviscous- and aerobactin-positive. It is important to improve the awareness towards the diagnosis and management of hvKp infections. Antibiotic resistance, biofilm formation and biofilm-related genes were studied to evaluate their relationships with one another and with the genetic genotypes and phenotypes of hvKp isolated from clinical settings. Analysis of our data showed that genes have a role in biofilm formation and antibiotic resistance patterns. These methods may help shed light on the connections between hvKp biofilm formation and antibiotic resistance, as well as the transmission pathways of clinical isolates. Further insight into the link between biofilm production and antibiotic resistance might aid in the fight against drug-resistant bacteria.

## Figures and Tables

**Table 1:** List of primers used in the study.

Gene		Primers Sequences	Reference
*rmpA*	Forward Reverse	5′-ACTGGGCTACCTCTGCTTCA-3′	[20]
		5′-CTTGCATGAGCCATCTTTCA-3′	
*rmpA2*	Forward Reverse	5′-CTTTATGTGCAATAAG-GATGTT-3′	[21]
		5′-CCTCCTGGAGAGTAAGCATT-3′	
*magA*	Forward Reverse	5′-GGTGCTCTTTACATCATTGC-3′	[20]
		5′-GCAATGGCCATTTGCGTTAG-3′	
*icuA*	Forward Reverse	5′-GCATAGGCGGATACGAACAT-3′	[10]
		5′-CACAGGGCAATTGCTTACCT-3′	
*K1*	Forward Reverse	5′-GTAGGTATTGCAAGCCATGC-3′	[19]
		5′-GCCCAGGTTAATGAATCCGT-3′	
*K2*	Forward Reverse	5′-GGAGCCATTTGAATTCGGTG-3′	[19]
		5′-TCCCTAGCACTGGCTTAAGT-3′	
*K5*	Forward Reverse	5′-GCCACCTCTAAGCATATAGC-3′	[19]
		5′-CGCACCAGTAATTCCAACAG-3′	
*K20*	Forward Reverse	5′-CCGATTCGGTCAACTAGCTT-3′	[19]
		5′-GCACCTCTATGAACTTTCAG-3′	
*K57*	Forward Reverse	5′-CGACAAATCTCTCCTGACGA-3′	[19]
		5′-CGCGACAAACATAACACTCG-3′	

**Table 2:** Virulence, microbiological and genetic characteristics of hvKp isolates.

No.	Specimen	Biofilm Formation	Serum Resistance	Capsule	rmpA	rmpA2	magA	iucA	String Test
1	Tracheal wash	Moderate	Resistant	K1	+	+	+	+	+
2	Tracheal wash	Moderate	Resistant	K2	+	+	+	+	+
3	Urine	Moderate	Resistant	K1	−	+	+	+	+
4	Wound	Strong	Intermediate sensitive	K1	+	−	−	+	+
5	Urine	Moderate	Resistant	-	+	+	+	+	+
6	Sputum	Moderate	Intermediate sensitive	K1	+	−	+	+	+
7	Urine	Strong	Intermediate sensitive	K2	+	−	+	+	+
8	Urine	Moderate	Resistant	K57	−	+	−	+	+
9	Sputum	Strong	Intermediate sensitive	K1	+	+	+	+	+
10	Urine	Moderate	Resistant	-	+	+	+	+	+
11	Tracheal wash	Strong	Resistant	K1	+	+	+	+	+
12	Tracheal wash	Moderate	Resistant	K2	+	+	+	+	+
13	Sputum	Strong	Intermediate sensitive	K1	+	+	+	+	+
14	wound	Moderate	Intermediate sensitive	K2	−	+	+	+	+
15	Urine	Strong	Intermediate sensitive	−	+	−	+	+	+
16	Sputum	Strong	Resistant	−	+	+	-	+	+
17	Urine	Strong	Intermediate sensitive	K1	+	−	+	+	+
18	Sputum	Strong	Intermediate sensitive	−	+	+	−	+	+
19	Urine	Strong	Intermediate sensitive	−	+	+	+	+	+

**Table 3:** The antimicrobial resistance pattern and ESBL production in hvKp vs. cKp.

Antimicrobial Agent	HvKp (n = 19) No. (%)	cKp (n = 101) No. (%)	*p*-Value
ESBL production	4 (21.05%)	51 (50.5%)	0.018
Amikacin	1 (5.3%)	10 (9.9%)	0.524
Gentamycin	2 (10.5%)	56 (55.4%)	<0.001
Ampicillin/clavulanic	4 (21%)	53 (52.5%)	0.022
Aztreonam	5 (26.3%)	44 (43.6%)	0.163
Cefepime	2 (10.5%)	51 (50.5%)	<0.001
Ceftriaxone	5 (26.3%)	71 (70.3%)	<0.001
Ceftazidime	5 (26.3%)	60 (59.4%)	0.002
Ciprofloxacin	7 (36.8%)	83 (82.2%)	<0.001
Levofloxacin	5 (26.3%)	60 (59.4%)	0.007
Trimethoprim/Sulfamethoxazole	2 (10.5%)	70 (69.3%)	<0.001
Piperacillin/Tazobactam	2 (10.5%)	53 (52.5%)	0.003
Imipenem	0 (00.00%)	2 (1.9%)	-
Meropenem	1 (5.3%)	5 (4.9%)	0.954
Ampicillin	19 (100%)	97 (96%)	0.613
Cefoxitin	4 (21%)	32 (31.7%)	0.4006

**Table 4:** Biofilm formation and serum resistance assay of non ESBL producing hvKP vs. non ESBL producing cKP.

	Non-ESBL-Producing hvKp (n = 15) No. (%)	Non-ESBL-Producing cKp (n = 50) No. (%)	*p*-Value
Biofilm Formation:			
Non/weak	0 (0%)	37 (74%)	<0.001
Moderate	7 (46.66%)	4 (8%)	0.018
Strong	8 (53.33%)	9 (18%)	0.043
Serum Resistance Assay:			
Sensitive	0 (0%)	38 (76%)	<0.001
Intermediate sensitive	8 (53.33%)	9 (18%)	0.043
Resistance	7 (46.66%)	3 (6%)	0.016

**Table 5:** Biofilm formation and serum resistance assay of non ESBL producing hvKp vs. ESBL-producing cKp.

	Non-ESBL-Producing hvKp (n = 15) No. (%)	ESBL-Producing cKp (n = 51) No. (%)	*p*-Value
Biofilm Formation:			
Non/weak	0 (0%)	10 (19.6%)	<0.001
Moderate	7 (46.66%)	8 (15.7%)	0.044
Strong	8 (53.33%)	33 (64.7%)	0.621
Serum Resistance Assay:			
Sensitive	0 (0%)	11 (21.6%)	<0.001
Intermediate sensitive	8 (53.33%)	8 (15.7%)	0.031
Resistance	7 (46.66%)	28 (55%)	0.542

**Table 6:** Biofilm formation and serum resistance assay of ESBL-producing hvKp vs. ESBL-producing cKp.

	ESBL-Producing hvKp (n = 4) No. (%)	ESBL-Producing cKp (n = 51) No. (%)	*p*-Value
Biofilm Formation:			
Non/weak	0 (0%)	10 (19.6%)	<0.001
Moderate	1 (25%)	8 (15.7%)	0.954
Strong	3 (75%)	33 (64.7%)	0.524
Serum Resistance Assay:			
Sensitive	0 (0%)	11 (21.6%)	<0.001
Intermediate sensitive	1 (25%)	8 (15.7%)	0.613
Resistance	3 (75%)	28 (55%)	0.452

**Table 7:** Differences between hvKp and cKp groups.

Characteristic	hvKp (n = 19) No. (%)	cKp (n = 101)	*p*-Value
Basic demographics:			
Age (mean ± SE)	31.26 ± 0.47	55.63 ± 1.43	<0.001
Male	10 (52.6%)	56 (56.4%)	0.822
K-serotype:			
K1	8 (42.1%)	3 (2.97%)	<0.001
K2	4 (21%)	2 (1.98%)	0.004
K5	0 (00.00%)	0 (00.00%)	-
K20	0 (00.00%)	0 (00.00%)	-
K57	1 (5.3%)	5 (4.95%)	0.954
Non typable	6 (31.6%)	91 (90.1%)	0.001
Virulence-associated Genes:			
rmpA	16 (84.2%)	16 (15.8%)	<0.001
rmpA2	14 (73.7%)	18 (17.8%)	<0.001
magA	15 (78.9%)	27 (26.7%)	0.037
Aerobactin	19 (100%)	6 (5.9%)	<0.001
Hypermucoviscosity:	19 (100%)	8 (7.9%)	<0.001
Underlying Diseases:			
Diabetes	15 (78.9%)	36 (35.6%)	0.003
Cancer	2 (10.5%)	13 (12.8%)	0.253
Pulmonary diseases	10 (52.6%)	44 (43.6%)	0.064
Chronic renal failure	0 (00.00%)	2 (1.9%)	-
Invasive Devices:	8 (42.1%)	5 (4.9%)	<0.001

## Data Availability

Available upon request.

## Abbreviations

Kp: *Klebsiella pneumoniae.* HAIs: hospital-acquired infections. cKp: classical *K. pneumoniae.* hvKp: *Hypervirulent Klebsiella pneumoniae.* PLA: pyogenic liver abscesses. MHC: major histocompatibility complex. hmvKp: hypermucoviscous *K. pneumonia. rmpA*: regulator of mucoid phenotype. *mag*A: mucoviscosity-associated gene. HA: hospital-acquired. ICU: intensive care unit. ESBL: extended-spectrum-β-lactamase. VCs: viable counts. PCR: polymerase chain reaction. SPSS: Statistical Package for Social Sciences.
